# Isolation and characterization of *Bacillus subtilis* strain 1-L-29, an endophytic bacteria from *Camellia oleifera* with antimicrobial activity and efficient plant-root colonization

**DOI:** 10.1371/journal.pone.0232096

**Published:** 2020-04-27

**Authors:** Jin-Xin Xu, Zi-Yang Li, Xing Lv, Hua Yan, Guo-Ying Zhou, Ling-Xue Cao, Qin Yang, Yuan-Hao He

**Affiliations:** 1 College of Forestry, Central South University of Forestry and Technology, Changsha, China; 2 Hunan Provincial Key Laboratory for Control of Forest Diseases and Pests, Central South University of Forestry and Technology, Changsha, China; 3 Key Laboratory for Non-wood Forest Cultivation and Conservation of Ministry of Education, Central South University of Forestry and Technology, Changsha, China; 4 Key Laboratory of National Forestry and Grassland Administration on Control of Artificial Forest Diseases and Pests in South China, Central South University of Forestry and Technology, Changsha, China; Universidade de Coimbra, PORTUGAL

## Abstract

Endophytic bacteria, which are common in plant tissues, may help to control plant pathogens and enhance plant growth. *Camellia oleifera*, an oil-producing plant, is widely grown in warm, subtropical, hilly regions in China. However, *C*. *oleifera* is strongly negatively affected by *C*. *oleifera* anthracnose, which is caused by *Colletetrichum fructicola*. To find a suitable biocontrol agent for *C*. *oleifera* anthracnose, 41 endophytes were isolated from the stems, leaves, and roots of *C*. *oleifera*. Bacterial cultures were identified based on analyses of 16S rDNA sequences; most strains belonged to the genus *Bacillus*. The antagonistic effects of these strains on *C*. *fructicola* were tested *in vitro*. In total, 16 strains inhibited *C*. *fructicola* growth, with *B*. *subtilis* strain 1-L-29 being the most efficient. Strain 1-L-29 demonstrated antagonistic activity against *C*. *siamense*, *C*. *asianum*, *Fusarium proliferatum*, *Agaricodochium camellia*, and *Pseudomonas syringae*. In addition, this strain produced indole acetic acid, solubilized phosphate, grew on N-free media, and produced siderophores. To facilitate further microecological studies of this strain, a rifampicin-resistant, green fluorescent protein (GFP)-labeled strain, 1-L-29*gfp*^r^, was created using protoplast transformation. This plasmid had good segregational stability. Strain 1-L-29*gfp*^r^ was re-introduced into *C*. *oleifera* and successfully colonized root, stem, and leaf tissues. This strain remained at a stable concentration in the root more than 20 d after inoculation. Fluorescence microscopic analysis showed that strain 1-L-29*gfp*^r^ thoroughly colonized the root surfaces of *C*. *fructicola* as well as the root vascular tissues of *Arabidopsis thaliana*.

## Introduction

Oil extracted from the seeds of the tea-oil camellia (*Camellia oleifera* Abel), which is rich in unsaturated fatty acids, vitamins, and various antioxidants, is commonly used in China for cooking [1]. Camellia oil is also used as a remedy for bowel, stomach, and burn-associated ailments in traditional Chinese medicine [2]. Thus, *C*. *oleifera* is widely commercially cultivated in many parts of China. *C*. *oleifera* anthracnose, a fungal infection caused by the *Colletetrichum gloeosporioides* species complex (CGSC) [3,4], is one of the most serious diseases affecting the tea-oil camellia [5–7]. This disease has severe detrimental effects, such as fruit drop, seed loss, or branch death, and may even lead to plant mortality [4].

Although chemical pesticides are a powerful and cost-effective method of anthracnose prevention, overuse of chemical pesticides might stimulate the development of pesticide-resistant fungal strains, and might have negative effects on human and environmental health [8]. Biocontrol methods, which utilize plant extracts and other biological agents, may be a promising alternative of anthracnose control. For example, endophytic bacteria may act as biocontrol agents, as these bacteria compete with bacterial pathogens.

Endophytes are plant-associated microorganisms that live in plant tissues without negatively affecting the plant host [9,10]. It has been shown that endophytic microorganisms may control plant pathogens [11–13], enhance plant growth [14], and improve phytoremediation. However, previous studies of endophytic bacteria have generally focused on model plants, including *Arabidopsis thaliana* [15], potatoes [16,17], and soybeans [18], as well as oil-producing trees, including olive trees [19,20], oil palms [21], and *Vernicia fordii* [22]. For example, the endophytic biocontrol strain *Pseudomonas fluorescens* PICF7 was extracted from olive trees; this bacterial strain was shown to interact with pathogens to induce a systemic defense response in the host tree; *P*. *fluorescens* PICF7 also colonizes and persists on or in wheat and barley root tissues [23–25]. Although endophytic bacteria in *C*. *oleifera* have been investigated, previous studies have focused on the identification of antagonistic bacteria. The community structures of endophytic bacteria in *C*. *oleifera* have not yet been studied.

Here, we aimed to address this knowledge gap by (i) characterizing the structure of the culturable endophytic microbial community in *C*. *oleifera*; (ii) evaluating the potential biocontrol applications of the endophytic bacteria based on their anti-pathogenic behaviors; and (iii) determining the growth-promotion potential and colonization capacities of these endophytic bacteria.

## Materials and methods

### Sample collection and isolation of endophytic bacteria

Leaf, stem, and root samples were collected from healthy tea-oil camellias growing in the Tianjilin Mountain, Experimental Base of Central South University of Forestry and Technology, Hunan Province, China (28°06′–28°07′ N, 113°02′–113°03′ E). Samples were surface-sterilized using a stepwise washing procedure [ethanol, 0.1% (w/v) mercuric chloride (HgCl_2_), and water] following Schulz et al. [26]. The sterilized samples were crushed in 5 ml of sterile distilled water for 30 min. Then, 50 μL of each suspension was plated onto nutrient agar (NA). In addition, 100 μL aliquots of the water from the final wash were plated onto NA to check the efficiency of sterilization. All plates, including the control, were incubated at 26–28°C for 7 d. Morphologically distinct colonies were selected and purified. Bacteria were grown in NA for 12 h at 28°C. Sterile glycerol was then added to the bacterial culture to a final concentration of 15%, and the bacterial-glycerol suspension was stored at −80°C until further analysis.

### DNA extraction, amplification, and sequencing

The endophytic bacterial isolates were grown in Luria-Bertani medium for 24 h at 28°C. Genomic DNA was extracted using Tiangen Bacterial Genomic DNA Extraction Kits (Tiangen, Beijing, China). To extract DNA, cells were lysed in three cycles of −80°C for 15 min and 37°C for 5 min. We PCR amplified the 16S rRNA gene in 25 μL reaction volumes, each containing 10 μL GoTaq Master mix (2X) (Promega, Wisconsin, USA), 1 μL of DNA template, and 0.2 μM of each primer (27F: 5’-AGAGTTTGATCCTGGCTCAG-3’ and 1492R: 5’-TACTTGTTACGACTT-3’). The PCR cycling condition were 5 min at 95°C; 35 cycles of 15 s at 94°C, 15 s at 50°C, and 90 s at 72°C; and 5 min at 72°C. PCR products were purified using Tiangen PCR Cleanup Kits, and sequenced on an ABI 3730 DNA Analyzer (ABI, CA, USA). Generated sequences were compared with public databases using NCBI BLASTN online (http://www.ncbi.nlm.nih.gov/). A phylogenetic tree was constructed based on this alignment using the neighbor-joining algorithm in MEGA 6.

### Identification of a highly antifungal bacterial endophyte

To quantify the antifungal activity levels of the endophytic bacterial taxa isolated from *C*. *oleifera* against *Colletetrichum fructicola in vitro*, the dual culture method was used [27]. We selected *C*. *fructicola* to represent the CGSC complex, as this species has a high isolation rate and is extremely virulent. In brief, for each endophyte strain, *C*. *fructicola* was inoculated in the middle of a potato dextrose agar (PDA) plate (d = 90 mm), and one endophyte was inoculated in an equilateral triangle approximately 3 cm from the pathogen. Control plates, inoculated with the pathogen but not the endophytic bacteria, were prepared in parallel. Plates were incubated for 7 d at 28°C, and then the diameters of the pathogen colonies were measured. The inhibition rate was then calculated as [(colony radius of the control group—colony radius of the test group) / colony radius of the control group] × 100%.

### Antagonistic activity of strain 1-L-29

Preliminary results showed that the endophyte identified as *B*. *subtilis* strain 1-L-29 had the highest level of antagonistic activity against *C*. *fructicola*. We repeated the dual culture method (following the methods described above) to measure the antifungal activity of strain 1-L-29 against two other members of the *C*. *gloeosporioides* complex, *C*. *siamense* and *C*. *asianum*, as well as three common fungal pathogens of *C*. *oleifera*: *Athelia rolfsii*, *Fusarium proliferatum*, and *Agaricodochium camellia* [28]. The antagonistic activity of strain 1-L-29 against a pathogenic plant bacterium, *Pseudomonas syringae* pv. Tomato D3000, was measured following the methods of Ghorbani [29].

The effects of strain 1-L-29 on the mycelial morphology of *C*. *fructicola* were observed under a microscope. Then, 10 μL of spore suspension (10^6^ spores/mL) was mixed with 20 μL of PDA medium containing 1 μL 1-L-29 (OD 0.8) on a sterile glass microscope slide. Each slide was placed under a sterile plastic petri dish with water-soaked filter paper. Plates were incubated at 28°C for 18 h.

### Physiological properties of strain 1-L-29

The nitrogen fixation capacity of strain 1-L-29 was estimated on nitrogen-free bromothymol blue (NFb) medium following Swamy *et al*. [30]; the inorganic phosphorus fixation capacity of this strain was estimated on Pikovskaia’s (PKO) medium following Li *et al*. [31]; and the organic phosphorus fixation capacity of this strain was estimated on Mongina organic culture medium following Schwyn and Neilands [32]. The siderophore production of strain 1-L-29 was measured on Chrom-Azurol Siderophore (CAS) agar medium following Rahman *et al*. [33]. Finally, the indole-3-acetic acid (IAA) production of strain 1-L-29 was estimated as previously described [34].

### Preparation of protoplasts

Strain 1-L-29 was incubated in LB liquid medium for 12 h at 37°C with shaking at Strain 1-L-29 was incubated in LB liquid medium by shaking at 200 rpm for 12 h at 37°C, and then 5 mL of this bacterial suspension was centrifuged at 4,000 rpm for 10 min at room temperature. The bacterial pellet was resuspended in 0.5 mL SMMP and lysozyme was added. SMMP medium was prepared by mixing equal volumes of 4X Penassay broth and 2X SMM (0.5 M sucrose, 0.02 M Maleate, and 0.02 M MgC12, pH 6.5 adjusted with NaOH). The cells were treated with 0.2, 5, 10, 20, 30, or 40 mg/mL lysozyme at 37°C with shaking at 120 rpm, and protoplast formation was determined after 0.5, 1, 1.5, and 2 h. Lysozyme was removed by centrifugation at 4,000 rpm for 5 min, followed by washing once in 2 mL of SMMP. The protoplasts were centrifuged again at 4,000 rpm for 5 min, resuspended in 0.5 mL of SMMP, diluted, and spread onto DM3 medium. Each liter of DM3 medium contained 200 mL of 4% agar, 500 mL of 1 M sodium succinate (pH 7.3), 100 mL of 5% casamino acids, 50 mL of 10% yeast extract, 100 mL of 3.5% K_2_HPO_4_ and 1.5% KH_2_PO_4_, 25 mL of 20% glucose, 20 mL of 1 M NaCl, and 5 mL of filter-sterilized 2% bovine serum albumin (added when the temperature of the mixture was ~55°C). Protoplasts were cultured on DM3 for 2–3 d at 37°C. The rate of protoplast formation was then calculated as (A − B) / A × 100%, where A and B were the number of colonies on the LB medium before and after lysozyme treatment, respectively.

### GFP labeling of strain 1-L-29

Strain 1-L-29 protoplasts were transformed following Guo [35]. Plasmid pGFP22 was constructed following Yao [36]. In brief, approximately 20 μL of pGFP22 and 20 μL of SMMP were thoroughly mixed into 0.5 mL of the protoplast SMMP suspension (protoplasts were produced under the optimal conditions determined above). The suspension was then incubated with 1.5 mL of 40% polyethylene glycol (PEG) 6000 in a 37°C water bath for 2 min. After incubation, 0.5 mL of SMMP was added to stop the reaction. The mixture was centrifuged at 4,000 rpm for 5 min, re-suspended in 1 mL of SMMP, and shaken at 60 rpm at 37°C for 2 h. Then, 0.1 mL of the suspension was spread onto DM3 medium containing 100 μg/mL ampicillin and cultured for 2–3 d at 37°C. Successful GFP labeling was confirmed with PCR. Plasmid pGFP22 DNA was purified using the Plasmid Mini Purification Kit. We PCR amplified the plasmid pGFP22 in 25 μL reaction volumes, each containing 10 μL GoTaq Master mix (2X) (Promega, WI, USA), 1 μL of DNA template, and 0.2 μM of each primer (*gfp*F: 5’-TAA GGG GGA AAT CAC ATG AGT AAA GGA GAA GAA-3’ and *gfp*R: 5’-GGG GTA CCA TTA TTT TTG ACA CCA GA-3’). The PCR cycling condition were 5 min at 95°C; 30 cycles of 30 s at 94°C, 30 s at 56°C, and 30 s at 72°C; and 5 min at 72°C. GFP-expressing bacteria were visualized using a Carl Zeiss AxioObserver A1 (Carl Zeiss, Jena, Germany) fluorescence microscope.

### Biological characteristics of strain 1-L-29*gfp*

To analyze growth curves, colonies of strain 1-L-29*gfp* and1-L-29 were inoculated into 100 mL of LB liquid medium, 1-L-29*gfp* medium supplemented with ampicillin, and cultured overnight at 37°C in a shaking incubator at 60 rpm. After incubation, 1 mL of each overnight culture was inoculated into 100 mL of ampicillin-containing LB, and cultured at 37°C in a shaking incubator at 150 rpm. The OD_600_ values of the two cultures were measured every 2 h to compare the growth curve of strain 1-L-29 to that of strain 1-L-29*gfp*.

The antifungal activity levels of strain 1-L-29*gfp* against *Colletetrichum fructicola*, *Colletetrichum siamense*, and *Colletetrichum asianum* were measured as described above.

The segregational stability of the plasmid was determined with a serial dilution culture method, using successive incubations. Specifically, strain 1-L-29*gfp* was diluted 1:1,000 in 5 mL antibiotic-free LB liquid medium and cultured for 60 h. Continual dilutions were performed during incubation, and 100 μL aliquots of the bacterial cultures were transferred every 5 h before dilution. Each aliquot was spread onto an antibiotic-free LB plate and cultured at 37°C overnight. Total colonies and GFP-positive colonies were counted under ultraviolet light. The segregational stability of the plasmid was represented by the number of GFP-positive colonies, as a percentage of the total number of colonies.

### The growth-promotion potential of 1-L-29*gfp*^r^

Strain 1-L-29*gfp*^r^, which was used in the inoculation, was grown in LB liquid medium for 24 h at 28°C. The cells were harvested by centrifugation at 4,000 rpm, and bacterial cell suspensions were prepared in sterile water (10^8^ CFU /mL). *C*. *oleifera* seeds were soaked in the bacterial suspension for 30 min, and then put on moistened absorbent cotton. Control seeds were soaked in distilled water. All treated *C*. *oleifera* seeds were maintained in a plant incubator (Panasonic, Ehime-ken, Japan) at 25°C with a 16-h light/8-h dark cycle. Various plant growth parameters, including fresh weight (FW), dry weight (DW), and root length were measured after 30 d of treatment. DW was measured after drying the samples in a hot air oven at 70°C for 72 h. This experiment was repeated three times.

### Colonization of *C*. *oleifera* by strain 1-L-29*gfp*^r^

Rifampicin-resistant strain 1-L-29*gfp* (designated strain 1-L-29*gfp*^r^) was produced following Glandorf [37]. In brief, rifampicin-resistant mutants of 1-L-29*gfp* were obtained by transferring colonies of this strain to LB medium agar plates containing increasing concentrations (5, 10, 20, 30, 50, 70, 100 μg/mL) of rifampicin. Strain 1-L-29*gfp*^r^, which showed the same antagonistic properties against *C*. *fructicola* as strain 1-L-29*gfp*, was selected.

We next tested whether 1-L-29*gfp*^r^ was able to colonize the roots of *C*. *oleifera*. In brief, strain 1-L-29*gfp*^r^ was diluted to 10^8^ CFU/mL using sterile water. The roots of two-year-old *C*. *oleifera* plants were soaked in the bacterial suspension for 20 min, and then transplanted into sterile soil. Positive controls and negative controls were soaked in the 1-L-29 bacterial suspension and in distilled water, respectively, before planting. Treated *C*. *oleifera* plants were kept at 25°C and 70% relative humidity with a 16-h light/8-h dark cycle. Endophytic bacteria were isolated from the roots, stems, and leaves of the treated plants at 0, 1, 3, 5, 7, 10, 15, 20, 25, and 30 d after treatment. Bacteria were isolated as described above, except that the nutrient agar medium was supplemented with 100 μg/mL rifampicin. This experiment was repeated three times.

### In planta visualization of strain 1-L-29*gfp*^r^

Strain 1-L-29*gfp*^r^ cells were washed twice in phosphate-buffered saline (1.44 g/L Na_2_HPO_4_, 0.24 g/L KH_2_PO_4_, 0.20 g/L KCl, 8.00 g/L NaCl; pH 7.4) (PBS) and resuspended in PBS (10^7^ cells/mL) prior to use. The roots of *C*. *oleifera* seedlings were then soaked in the 1-L-29*gfp*^r^ bacterial suspension for 20 min at 28°C. Control plants were soaked in sterilized PBS buffer under the same conditions. All seedlings were transplanted into sterile soil and maintained at 28°C with a 16-h light/8-h dark cycle. Plants were carefully uprooted from pots, and root systems were washed by dipping in tap water. Root samples were exposed to 10 cycles of 30 sec ultrasound treatments. Plant roots were sampled at 0, 24, 48, and 72 h after bacterial inoculation, and GFP fluorescence was visualized using a Carl Zeiss AxioObserver A1 fluorescence microscope with a 450–490 nm excitation filter. To observe whether the colonization mechanisms of strain 1-L-29 in *C*. *oleifera* differed from those in other plants, we studied the colonization of *Arabidopsis thaliana* roots by strain 1-L-29*gfp*^r^ as described above for *C*. *oleifera*.

### Data analysis

We performed analyses of variance (ANOVAs) on all data. Multiple comparisons of test data were implemented using Duncan’s new multiple range test at a 5% probability level, using SPSS version 16.0 (SPSS Inc., IL, USA).

## Results

### Identification of endophytic bacterial isolates

We isolated, purified, and cultured 41 bacterial species from healthy *C*. *oleifera* leaves (10 species; 24.4%), roots (23 species; 56.1%), and stems (8 species; 19.5%). The lack of bacterial colonies on control plates indicated that the isolates obtained were endophytic. Based on our 16S rRNA phylogeny, all isolated bacterial taxa fell into the genera *Bacillus*, *Sporosarcina*, *Paenibacillus*, *Achromobacter*, or *Enterobacter* (Table 1; S1 Fig).

**Table 1:** Endophytic bacteria isolated from surface-sterilized *C*. *oleifera*.

Isolates	GenBank Accession No. ^a^	Closest phylogenetic relative (GenBank Accession No.)	Identity (%)
Isolates from leaves			
1-L-28	MK133136	*B*. *amyloliquefaciens* (KY685067.1)	99
1-L-26	MK133133		99
1-L-24	MK133122		99
1-L-32	MK133119		99
1-L-1	MK133120		99
1-L-28	MK133123		99
3-L-2	MK133125		99
1-L-29	MK133134	*B*. *subtilis* (MF957285.1)	99
1-L-27	MK133132		99
1-L-21	MK133128		99
Isolates from stems			
1-S-25	MK133126	*B*. *amyloliquefaciens* (KY685067.1)	99
2-S-2	MK133124		99
1-S-15	MK133121		99
1-S-12	MK133130		99
2-S-1	MK133127		99
1-S-22	MK133131	*A*. *xylosoxidans* (LC125142.1)	99
1-S-2	MK133129		99
2-S-3	MK133102	*Paenibacillus sp*. (JN617220.1)	99
Isolates from roots			
1-R-2	MK133101	*B*. *cereus* (KJ524505.1)	99
1-R-4	MK133103		99
1-R-6	MK133105		99
1-R-7	MK133106		99
2-R-1	MK133107		99
2-R-2	MK133108		98
2-R-3	MK133109		96
2-R-4	MK133110		96
2-R-7	MK133113		99
1-R-9	MK133117		98
1-R-10	MK133118		99
1-R-1	MK133100	*Enterobacter sp*. (MH725605.1)	99
1-R-5	MK133104		99
2-R-5	MK133111		99
2-R-6	MK133112		97
2-R-8	MK133114		99
2-R-9	MK133115		97
1-R-11	MK133137	*E*. *cloacae* (CP022148.1)	99
1-R-12	MK133138		99
1-R-13	MK133139		99
1-R-14	MK133140		99
2-R-10	MK133116	*S*. *luteola* (KP100329.1)	99
1-R-8	MK133135	*B*. *amyloliquefaciens* (KY685067.1)	99

### Anti-pathogenic and physiological properties of the endophytic bacteria

**Fig 1:**
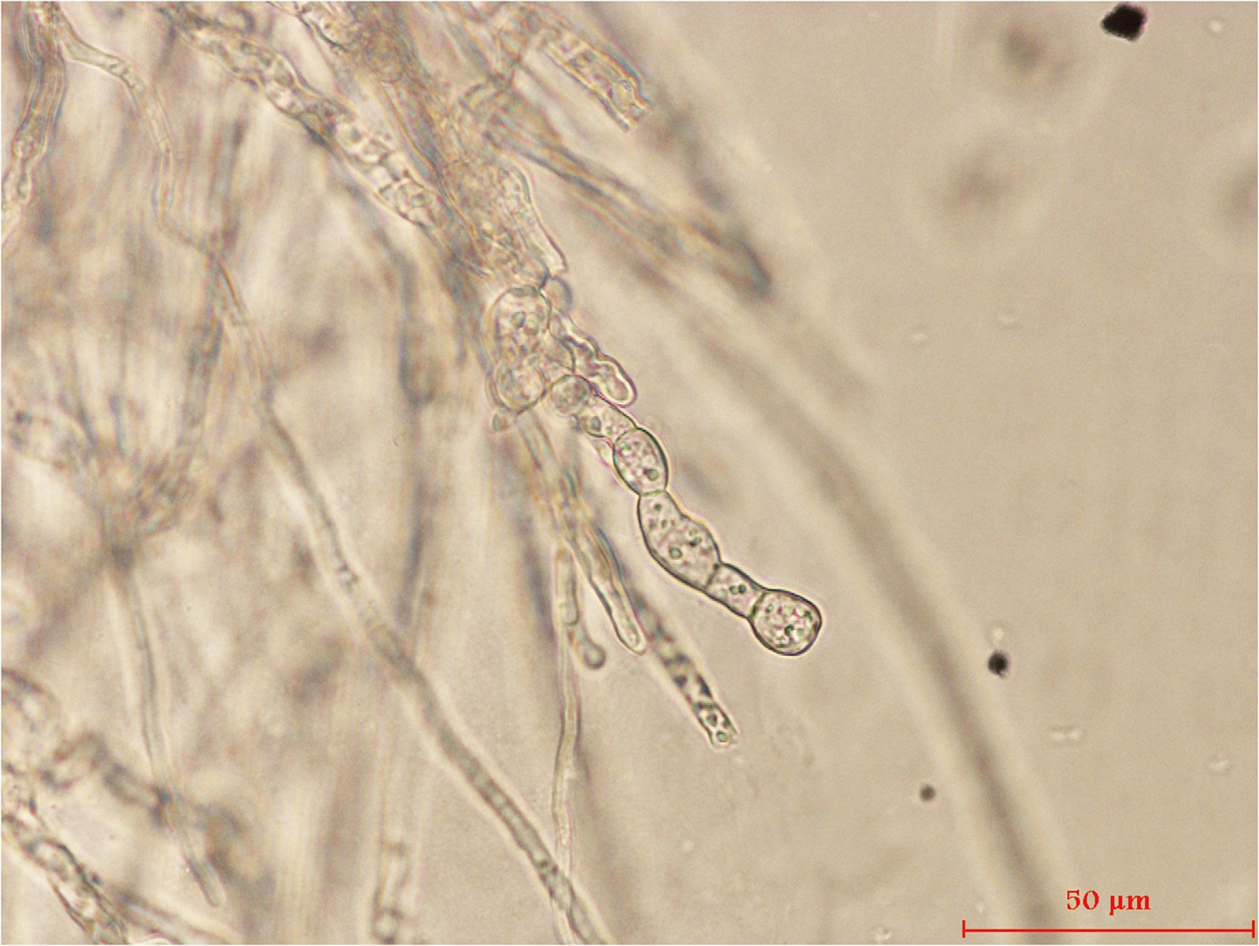
Mycelial morphology of *C*. *fructicola* after exposure to *B*. *subtilis* strain 1-L-29.

Our dual culture analyses showed that 16 endophytic bacterial strains (of 41 total) exhibited antifungal properties (Table 2). Each of these 16 strains competed with *C*. *fructicola* for nutrients or space, or otherwise inhibited the mycelial growth of this fungus. Strain 1-L-29 had the highest inhibition rate against *C*. *fructicola* (although not significantly higher than several other strains; Table 2). In addition, micrographs showed that exposure to strain 1-L-29 led to hyphal deformation in *C*. *fructicola*, as well as the enlargement of the cytoplasmic vacuoles (Fig 1). For these reasons, strain 1-L-29 was chosen for further analyses. We found that strain 1-L-29 inhibited *C*. *siamense*, *C*. *asianum*, *F*. *proliferatum*, *A*. *camellia*, and *P*. *syringae*, but not *A*. *rolfsii* (Table 3).

**Table 2:** Inhibition of *C*. *fructicola* mycelial growth by endophytic bacteria from *C*. *oleifera*.

Endophyte strains	Pathogenic colony radius (mm)	Pathogen growth inhibition rate (%)
1-L-29	22.33 ± 4.51 ab	(68.1 ± 6.44) a
1-L-27	26 ± 4 a	62.86 ± 5.71 ab
1-R-8	28.67 ± 3.51 a	(59.05 ± 5.02) b
2-S-3	27.67 ± 1.53 ab	(60.48 ± 2.18) ab
1-S-22	28.33 ± 3.21 ab	(59.52 ± 4.59) b
1-L-26	27.47 ± 2.2 ab	(60.76 ± 3.15) ab
1-L-1	23.33 ± 3.06 ab	(66.67 ± 4.36) ab
1-L-32	25.2 ± 2.43 ab	(64 ± 3.48) ab
1-S-15	27.33 ± 2.31 ab	(60.95 ± 3.3) ab
1-S-12	25.67 ± 3.21 ab	(63.33 ± 4.59) ab
2-S-2	26.33 ± 4.04 ab	(62.38 ± 5.77) ab
1-L-21	22.67 ± 4.62 ab	(67.62 ± 6.6) ab
2-S-1	24.67 ± 1.15 ab	(64.76 ± 1.65) ab
1-L-28	24.67 ± 4.62 ab	(64.76 ± 6.6) ab
1-L-24	26.8 ± 1.39 b	(61.71 ± 1.98) ab
1-S-25	26 ± 2 b	(62.86 ± 2.86) ab
CK	70.31± 1.71	0

Data shown are mean ± standard error of three replicates. Different lowercase letters within the same column indicate significant differences (p < 0.05). CK: Control *C*. *fructicola* plates, without endophytic strains.

**Table 3:** Antagonistic activity of strain 1-L-29 against various pathogens.

Pathogen	Antagonistic effect
*C*. *siamense*	Positive
*C*. *asianum*	Positive
*A*. *rolfsii*	Negative
*F*. *proliferatum*	Positive
*A*. *camellia*	Positive
*P*. *syringae*	Positive

Strain 1-L-29 produced IAA, grew on N-free media, and solubilized both organic and inorganic phosphorus. When plated on CAS medium, strain 1-L-29 produced a zone of yellowish-orange color, indicating the production of siderophores.

### Construction and verification of GFP-labeled strain 1-L-29*gfp*

**Fig 2:**
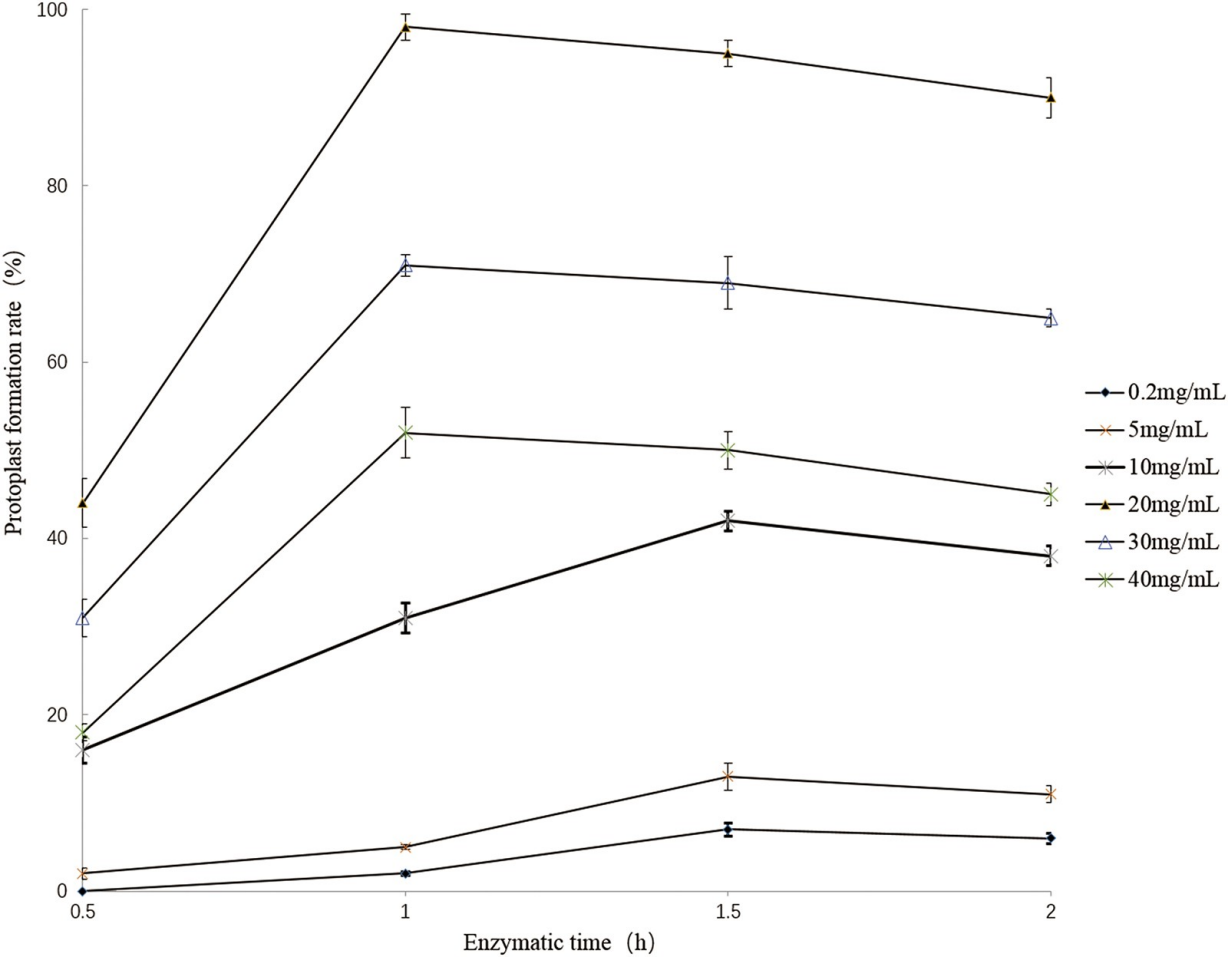
Effects of lysozyme concentration and digestion time on the protoplast formation of *Bacillus subtilis* strain 1-L-29.

The protoplast formation rate for strain 1-L-29 was greatest (98%) after 1 h of digestion with 20 mg/mL lysozyme (Fig 2). Therefore, these conditions were used to produce protoplasts from strain 1-L-29.

**Fig 3:**
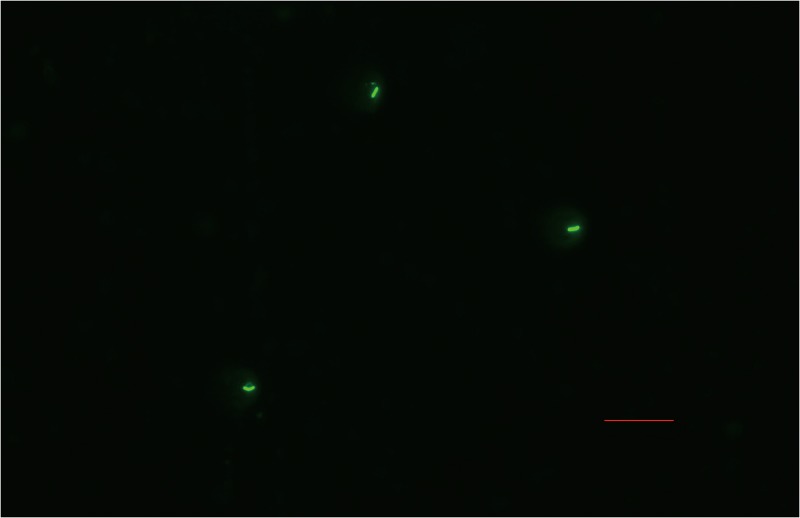
Cells of *Bacillus subtilis* strain 1-L-29*gfp* under a fluorescence microscope (40 × magnification).

**Fig 4:**
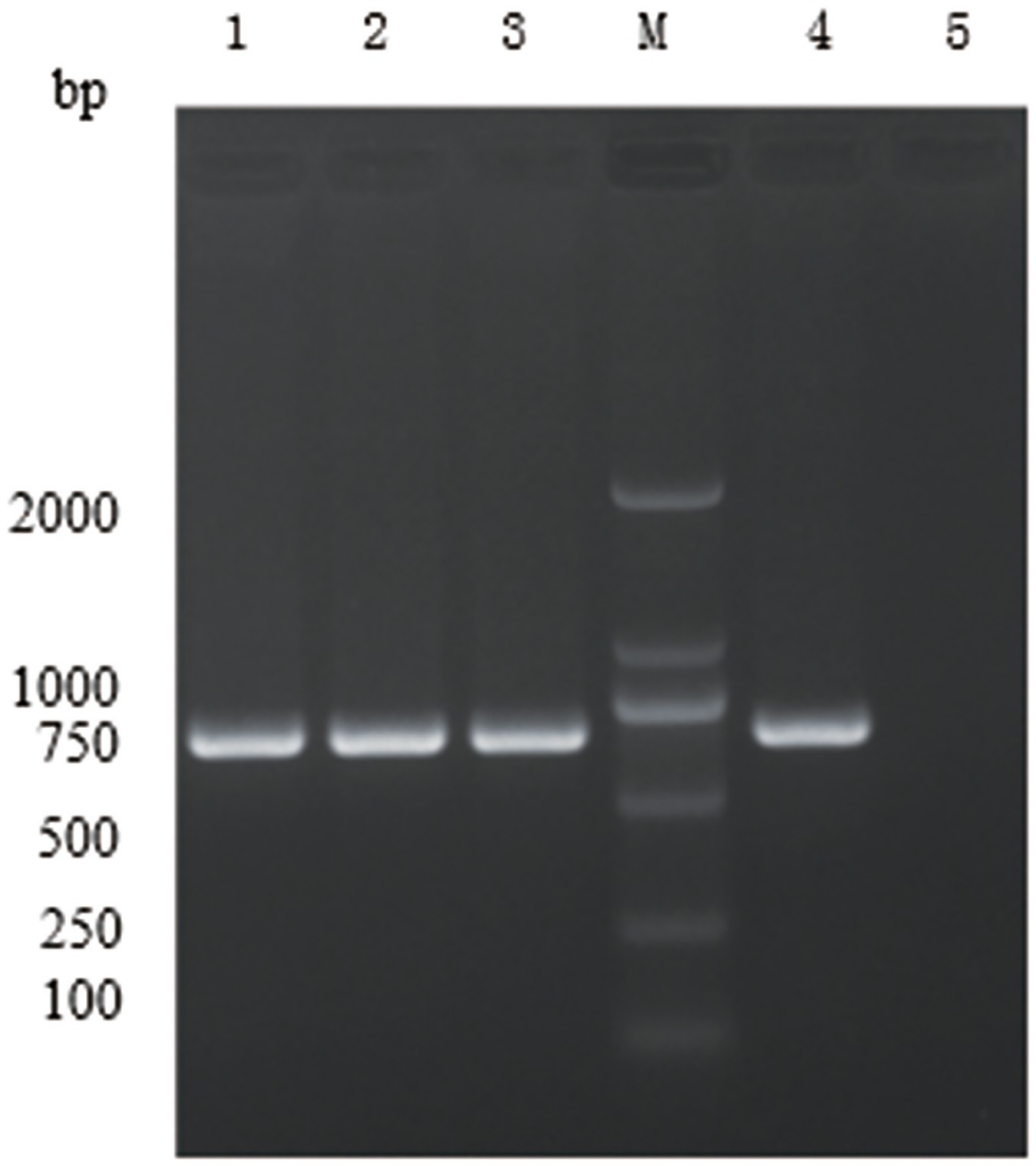
Electrophoresis of PCR products. Lane M: DNA marker; lanes 1–3: genomic DNA of strain 1-L-29*gfp*; 4: plasmid pGFP22; 5: genomic DNA of strain 1-L-29.

Strain 1-L-29 transformed with the recombinant expression vector pGFP22 fluoresced green (Fig 3), and the expected 750 bp PCR product was amplified using GFP-specific primers (Fig 4). This indicated that the GFP-labeled strain 1-L-29 (strain 1-L-29*gfp*) had been successfully constructed.

The growth curves of wild-type strain 1-L-29 and GFP-labeled strain 1-L-29*gfp* were similar (S2 Fig): both strains reached the logarithmic growth phase after 2 h of culture, the late stage of logarithmic growth after 10 h, and then entered the stationary phase. Thus, the presence of the plasmid pGFP22 and the expression of GFP did not noticeably affect the growth of 1-L-29. The inhibition rates of 1-L-29*gfp* against *C*. *fructicola*, *C*. *siamense* and *C*. *asianum* did not differ significantly from those of 1-L-29 (S1 Table). After 10 generations, the plasmid segregational stability of 1-L-29*gfp* was 88% in continual dilution culture, and 91% after successive incubations (S3 Fig).

### The growth-promotion potential of 1-L-29*gfp*^r^

Root length, fresh weight, and dry weight were significantly greater in 1-L-29*gfp*^r^-inoculated *C*. *oleifera* plants than in control plants (Table 4). In 1-L-29*gfp*^r^-inoculated seedlings, root length increased by 49.3%, root fresh weight increased by 62.9%, and root dry weight increased by 77.7% as compared to control seedlings.

**Table 4:** Effects of 1-L-29*gfp*^r^ on root length, fresh weight, and dry weight.

Treatment	Root length (cm)	Root fresh weight (g)	Root dry weight (g)
1-L-29*gfp*^r^	7.27±0.41 a	0.589±0.045 a	0.192±0.014 a
CK	4.87±0.33 b	0.365±0.019 b	0.108±0.014 b

Data shown are mean ± standard error of three replicates. Different lowercase letters within the same column indicate significant differences (p < 0.05).

### Colonization of *C*. *oleifera* by strain 1-L-29*gfp*^r^

The mutant strain 1-L-29*gfp*^r^ was resistant to 100 μg/mL rifampicin. There were no obvious differences in morphology or antifungal activity between strain 1-L-29*gfp* and strain 1-L-29*gfp*^r^. Colonization of *C*. *oleifera* by strain 1-L-29*gfp*^r^ peaked on day 0 after inoculation in the root, and on day 1 after inoculation in the stem and leaf (Table 5). Colonization steadily decreased in all plant tissues after this point, plateauing after day 20.

**Table 5:** Colonization of *C*. *oleifera* after a single inoculation with *B*. *subtilis* strain 1-L-29*gfp*^r^.

Treatments	Day post-inoculation	Colonization (10^3^ CFU/g)		
		Root	Stem	Leaf
1-L-29*gfp*^r^	0	17.8 ± 0.97 a	0 ± 0 f	0 ± 0 h
	1	11.97 ± 0.39 b	6.01 ± 0.17 a	3.06 ± 0.09 a
	3	8.62 ± 0.37 c	2.33 ± 0.13 b	1.32 ± 0.06 b
	5	5 ± 0.73 d	1.3 ± 0.19 c	0.83 ± 0.07 c
	7	2.16 ± 0.31 e	0.66 ± 0.08 d	0.5 ± 0.03 d
	10	1.9 ± 0.04 ef	0.35± 0.06 df	0.34 ± 0.06 e
	15	1.42 ± 0.09 efg	0.21± 0.03 df	0.25 ± 0.05 f
	20	1.18 ± 0.03 fg	0.13± 0.05 df	0.16 ± 0.02 g
	25	0.73 ± 0.03 g	0.11± 0.05 df	0.14 ± 0.02 g
	30	0.64 ± 0.04 g	0.1 ± 0.05 df	0.13 ± 0.02 g
Positive control	0–30	-	-	-
Negative control	0–30	-	-	-

Positive controls were soaked in the 1-L-29 bacterial suspension; Negative controls were soaked in distilled water. -, no bacteria. Data shown are mean ± standard error of three replicates. Different lowercase letters within the same column indicate significant differences (p < 0.05).

### In planta visualization of strain 1-L-29*gfp*^r^

**Fig 5:**
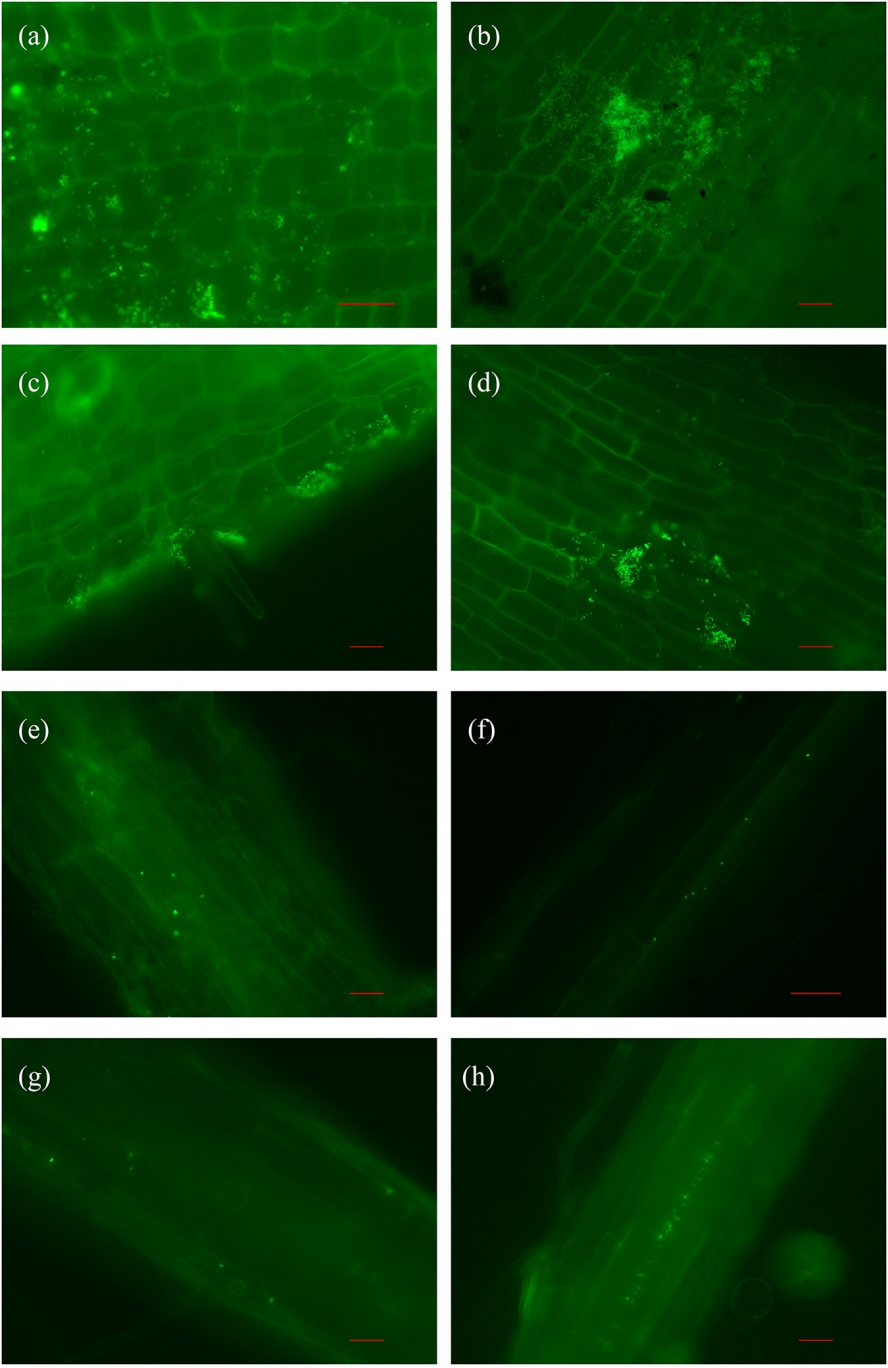
Colonization of the roots of (a–d) *C*. *oleifera* and (e–h) *A*. *thaliana* by *B*. *subtilis* strain 1-L-29*gfp*^r^ (300 × magnification). Colonization after (a) 0 h; (b, e, f) 24 h; (c) 48 h; and (d, g, h) 72 h is indicated by green fluorescence. Scale bars = 50 μm.

GFP-expressing cells were visible at high densities in the roots of *C*. *oleifera* and *A*. *thaliana*, clearly demonstrating successful colonization (Fig 5). At 24 h post-inoculation, many 1-L-29*gfp*^r^ cells were distributed uniformly on the surfaces of the *C*. *oleifera* roots (Fig 5a). However, bacterial numbers decreased between 24 and 72 h post-inoculation, and few bacteria were observed on the root surfaces of *C*. *oleifera* at 72 h post-inoculation (Fig 5g). Colonies of 1-L-29*gfp*^r^ cells were also observed on the root surfaces and in the vascular tissues of *A*. *thaliana*. In contrast to *C*. *oleifera*, few bacteria were observed on the root surfaces of *A*. *thaliana*. In the vascular tissues of *A*. *thaliana*, more 1-L-29*gfp*^r^ cells were observed after 72 h (Fig 5h) then after 24 h (Fig 5f).

## Discussion

Although *C*. *oleifera* is currently of major agricultural importance in southern China as the source of tea oil, farmers do not manage this plant intensively, and may apply fertilizer only once or not at all. Therefore, it is important to identify a microorganism that can effectively colonize *C*. *oleifera*, prevent plant disease, and improve soil fertility. Endophytic bacteria are widely used as biocontrol agents as they can successfully colonize target plants. The endophytic bacteria of *C*. *oleifera* have been studied to some extent. For example, He isolated and screened antagonistic endophytic bacteria, aiming to control *C*. *oleifera* anthracnose [38]. However, the pathogen identified in this previous study, *C*. *gloeosporioides*, was later shown not to be the primary pathogen causing *C*. *oleifera* anthracnose [4]. Here, we identified microbes that inhibited the primary pathogen causing *C*. *oleifera* anthracnose, *C*. *fructicola*. The identified bacterial strains also solubilized phosphate, grew on N-free media, and produced siderophores. These properties rendered the identified strains suitable for the soil environment of Hunan, China, and for the management of *C*. *oleifera*.

The endophytic bacteria of *C*. *oleifera* were studied using cultivation methods. Most of these bacteria were obtained from the root. This suggested that roots are appropriate habitats for endophytic bacteria. The 41 endophytic bacteria identified in *C*. *oleifera* fell into the classes Bacilli, γ-Proteobacteria, and β-Proteobacteria. Of the identified strains, *B*. *subtilis* strain 1-L-29 strongly inhibited *C*. *fructicola*, as well as several other plant pathogens. Culturable endophytic *Bacillus* species are frequently isolated from soils and plant tissues, including rhizospheres and leaves [39–42]. Due to the spore-forming abilities of these bacteria, they are highly resistant to adverse ecological conditions. Many *Bacillus* strains promote plant growth and/or produce a wide variety of antibiotic metabolites; as such, *Bacillus* strains are often used for the biocontrol of plant diseases [43].

Here, strain 1-L-29 negatively affected the mycelial growth and morphology of *C*. *fructicola* without direct contact, possibly because this strain produces antifungal chemicals or emits antifungal volatile organic compounds. Similarly, exposure to the crude culture filtrate extract of *Streptomyces* sp. MJM5763 inhibited spore germination in *C*. *gloeosporioides* and greatly altered mycelial morphology, causing swelling, excessive branching with large vesicles, and stunted hyphal growth [44]. In addition, treatment with *B*. *amylolicefaciens* reduced *C*. *lindemuthianum* spore numbers and inhibited mycelial growth [45]. *B*. *subtilis* 1-L-29 inhibited *C*. *fructicola*, *C*. *siamense*, *C*. *asianum*, *F*. *proliferatum*, *A*. *camellia*, and *P*. *syringae*, but not *A*. *rolfsii* (Table 3). *Bacillus* species, which are considered good sources of molecules with antimicrobial activity, produce well-known substances such as bacitracin, bacteriocins, and antimicrobial lipopeptides. *B*. *subtilis* strains also produce volatile organic compounds (VOCs), some of which promote plant growth and/or activate plant defense mechanisms by triggering systemic resistance. Interestingly, our preliminary tests indicated that volatile substances have inhibitory effects on *C*. *fructicola* mycelium growth and pigmentation (results not shown).

Strain 1-L-29 might also improve plant growth. That is, the acidic soils in which *C*. *oleifera* is grown in southern China commonly have low levels of available phosphorus; this may limit tea-oil yield [46]. As our results indicated that strain 1-L-29 solubilized organic and inorganic phosphorus, treatment with this strain might increase the amount of phosphorus available to *C*. *oleifera*, enhancing plant growth. Endophytic bacteria can also promote plant growth by producing the phytohormone IAA [47]. IAA increases root size and distribution, resulting in greater nutrient absorption from the soil. Although we found that strain 1-L-29 produced IAA, we did not quantify the concentration of IAA produced. To test whether strain 1-L-29 improved growth, *C*. *oleifera* seeds were soaked in a bacterial suspension. Subsequent phenotypic evaluation showed that bacterial exposure increased root length, fresh weight, and dry weight. This suggested that strain 1-L-29 might promote growth when used to treat *C*. *oleifera* seeds.

Strain 1-L-29 might be useful for biocontrol and growth-promotion applications if this strain successfully colonizes *C*. *oleifera*. To test this, it was necessary to tag the strain. Although previous studies have primarily used antibiotic markers, such as rifampicin, ampicillin, and kanamycin, to label endophytic bacteria, this method cannot distinguish the labeled strain from microorganisms with natural antibiotic resistance [48]. Labeling with GFP overcomes this problem. Typically, marker genes are introduced into *B*. *subtilis* strains using natural competence, protoplast transformation, or electroporation [49]. Electroporation, which is efficient, simple, and widely applicable, is the most commonly-used transformation method [49]. However, a higher voltage is required to penetrate *Bacillus* cell walls as compared to the cell walls of *Escherichia coli*, and this higher voltage often kills the target cell. Therefore, the preparation of electrocompetent *Bacillus* cells is complicated, and protoplast transformation efficiency is low. In this study, we attempted electroporation with strain 1-L-29 under a variety of conditions but were unsuccessful. We thus used the protoplast method, and successfully constructed a GFP-tagged strain.

Strains 1-L-29*gfp* and 1-L-29 had similar rates of growth and levels of antipathogenic activity, suggesting that exogenous plasmids had no significant effects on bacterial growth and antagonistic activity.

Strain 1-L-29 was labeled with the *gfp*-containing plasmid. The segregational stability of this the plasmid is generally low. However, we found that 91% of all 1-L-29*gfp* cells contained the recombinant plasmid after 60 h of serial dilution culture. If we assume that the doubling time of 1-L-29*gfp* is 20 min in a serial dilution culture, then the 1-L-29*gfp* cells would have divided approximately 180 times in 60 h, implying a plasmid loss frequency of about 5 × 10^−4^/generation. The plasmid segregational stability of strain 1-L-29*gfp* under continuous culture conditions was higher than that of this strain under continuous dilution culture conditions. This might be because the engineered strain was prone to spore formation due to nutrient limitation in the continuous culture, and plasmids were easily lost during spore formation. This suggested that 1-L-29*gfp* required additional nutrients. To obtain high stability and to prevent genetic burden from affecting growth of the labeled strain, a single copy of the *gfp*^+^ gene could be integrated into the bacterial chromosome. However, the expression of the single *gfp*^+^ copy in the bacterial genome would result in a relatively low intensity of green fluorescence; this is especially problematic in G^+^ bacteria with cell envelopes consisting of multiple peptidoglycan layers [50]. Therefore, bacteria with a low intensity of green fluorescence might be barely distinguishable from auto-fluorescent plant tissues. Therefore, a plasmid was chosen to carry the *gfp* gene.

Our colonization experiments showed that the density of 1-L-29*gfp*^r^ inside *C*. *oleifera* roots and leaves was greater than 0.13 × 10^3^ CFU/g (Table 5). In addition, 1-L-29*gfp*^r^ colonized the root epidermal surfaces (Fig 5), suggesting endophytic colonization. Strain 1-L-29*gfp*^r^ colonized the roots, stems, and leaves of *C*. *oleifera*, but 1-L-29*gfp*^r^ density was typically higher in the root. In general, populations of introduced endophytic bacteria remain stable at 10^3^–10^5^ CFU/g in the roots of most plant species investigated to date [51]. However, due to the long treatment time (30 d), the densities of 1-L-29*gfp*^r^ were lower than have been reported previously. Although bacteria were isolated from the leaves of *C*. *oleifera*, the densities of the 1-L-29*gfp*^r^ colonies in the roots were greater than the densities of these colonies in the leaves. The explanation for this is unclear, but it is possible that the leaf bacteria we isolated entered the plant through the roots and were then transported to the leaves.

The fluorescence microscope images showed that strain 1-L-29*gfp*^r^ successfully colonized the roots of *C*. *oleifera*, which is a woody plant. In the vascular tissues of *A*. *thaliana*, more 1-L-29*gfp*^r^ cells were observed after 72 h (Fig 5h) then after 24 h. This indicated that the strain 1-L-29*gfp*^r^ also successfully colonized, and was transported throughout, the herbaceous plant *A*. *thaliana*. Thus, strain 1-L-29 might be useful as a biocontrol agent in herbaceous plants, as well as in woody plants.

In *C*. *oleifera*, strain 1-L-29*gfp* extensively colonized the root surfaces, forming aggregates. These aggregations were not present in *A*. *thaliana*, possibly because plants selectively recruit beneficial rhizobacteria [52].

In summary, our results showed that the endophytic *B*. *subtilis* strain 1-L-29, isolated from *C*. *oleifera*, might have broad applications as a biocontrol and growth-promotion agent in both woody and herbaceous plants. Our results may be particularly useful for the biological control of *C*. *oleifera* anthracnose.

## Supporting information

S1 Fig Phylogenetic tree based on partial 16S rRNA sequences from bacterial strains isolated from *Camellia oleifera*, as well as those of selected reference strains (see Table 1 for details). (TIF)

S2 Fig Growth curves of the endophytic bacteria *Bacillus subtilis* 1-L-29 and 1-L-29*gfp*. (TIF)

S3 Fig Plasmid segregational stability of the green fluorescent protein (GFP)-tagged mutant *Bacillus subtilis* strain 1-L-29*gfp*. (TIF)

S1 Table The inhibitory effects of *Bacillus subtilis* strain 1-L-29*gfp* and strain 1-L-29 on various fungal species. (XLSX)

S1 Raw images (PDF)
