# Supplementary figures and images for: Isolation and characterization of Bacillus subtilis strain 1-L-29, an endophytic bacteria from Camellia oleifera with antimicrobial activity and efficient plant-root colonization
Source: PLoS One. 2020 Apr 27;15(4):e0232096. doi: 10.1371/journal.pone.0232096 (PMC7185607; doi:10.1371/journal.pone.0232096)

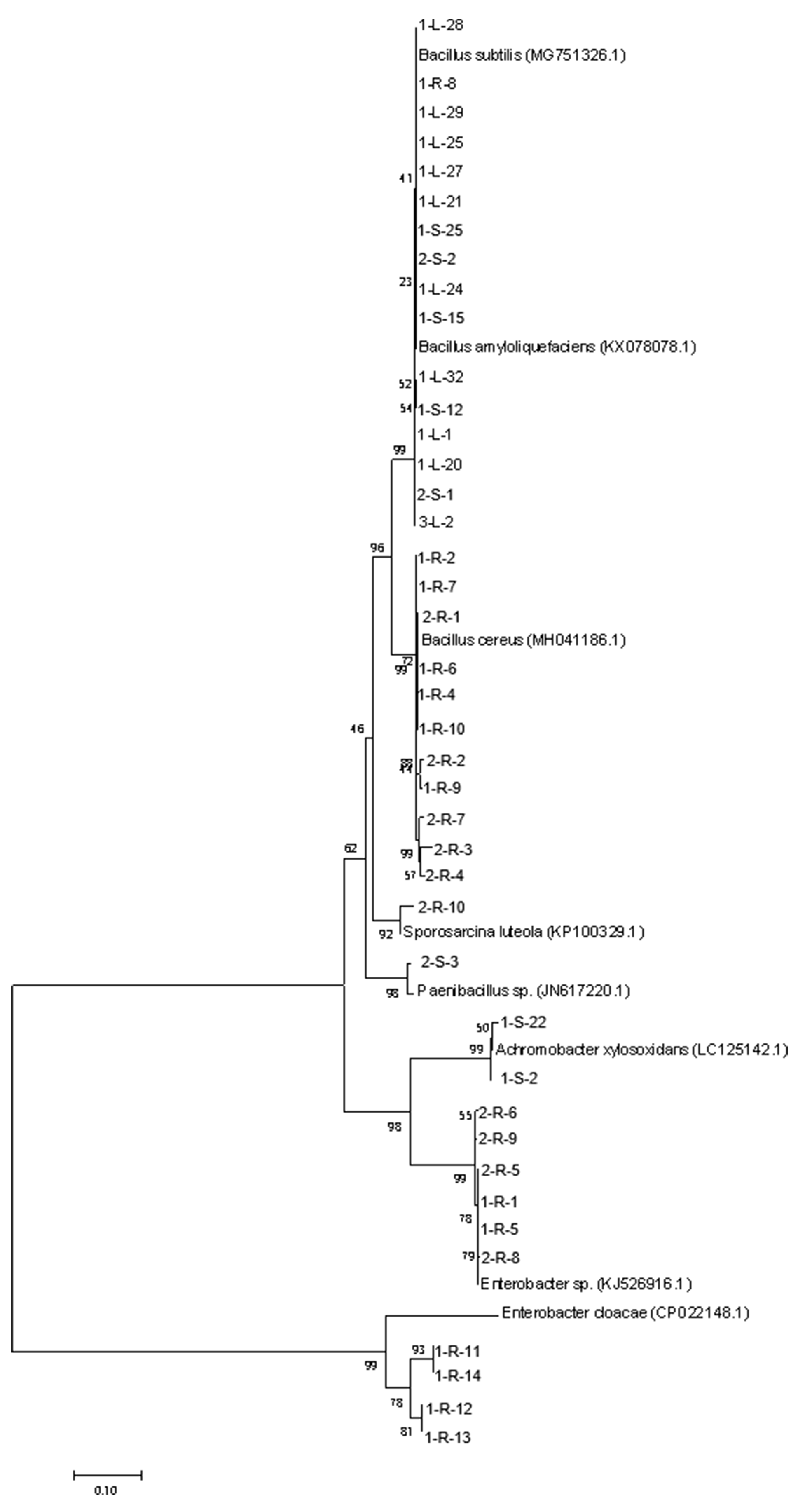

Supplement: S1 Fig — (TIF) [file pone.0232096.s001.tif]

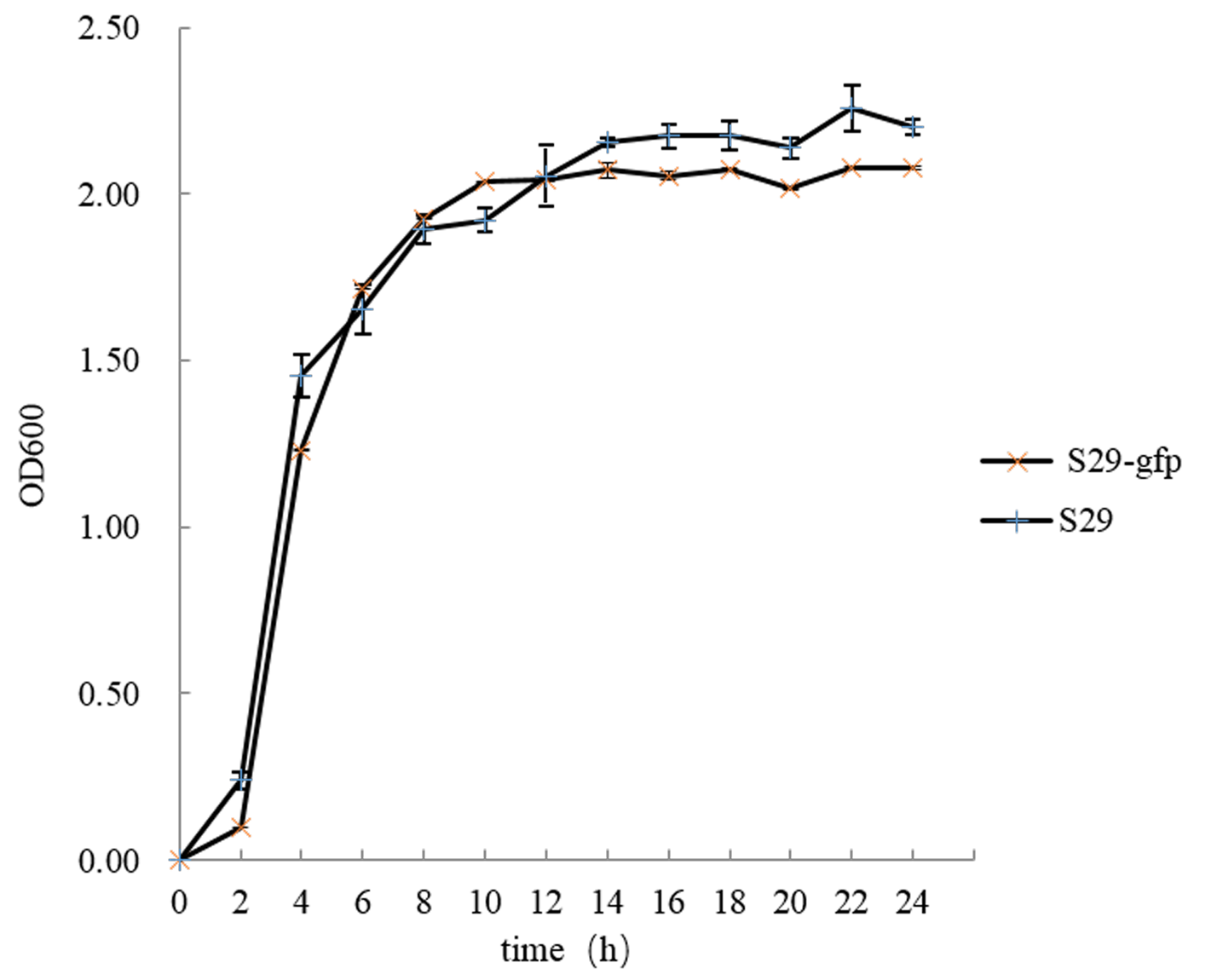

Supplement: S2 Fig — (TIF) [file pone.0232096.s002.tif]

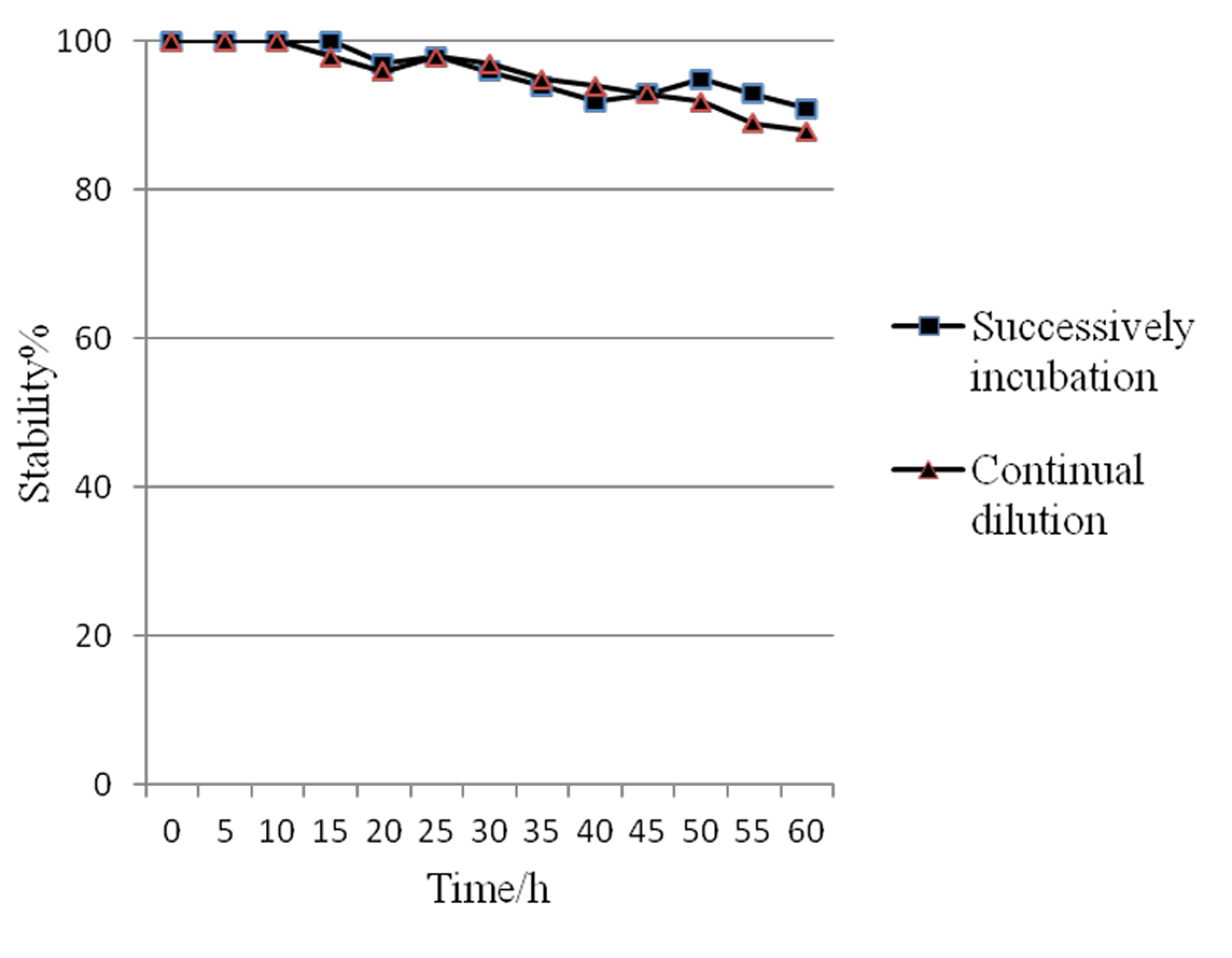

Supplement: S3 Fig — (TIF) [file pone.0232096.s003.tif]

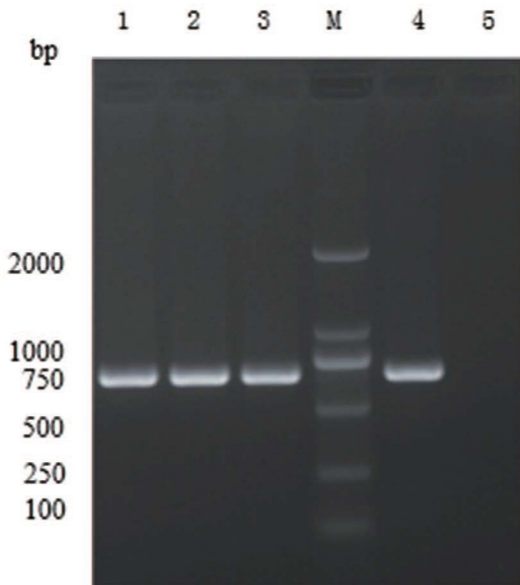

Supplement: S1 Raw images — (PDF) [file pone.0232096.s005.pdf]
